# Spray-Dried Cytisine-Loaded Matrices: Development of Transbuccal Sustained-Release Tablets as a Promising Tool in Smoking Cessation Therapy

**DOI:** 10.3390/pharmaceutics14081583

**Published:** 2022-07-29

**Authors:** Giuseppe Angellotti, Giulia Di Prima, Amalia Giulia Scarpaci, Fabio D’Agostino, Giuseppina Campisi, Viviana De Caro

**Affiliations:** 1 Dipartimento di Discipline Chirurgiche, Oncologiche e Stomatologiche, Università degli Studi di Palermo, Via L. Giuffrè 5, 90127 Palermo, Italy; giuseppe.angellotti@unipa.it; 2 Dipartimento di Scienze e Tecnologie Biologiche Chimiche e Farmaceutiche (STEBICEF), University of Palermo, Via Archirafi 32, 90123 Palermo, Italy; amaliagiulia.scarpaci@unipa.it (A.G.S.); viviana.decaro@unipa.it (V.D.C.); 3 Istituto per lo Studio degli Impatti Antropici e Sostenibilità dell’Ambiente Marino, Consiglio Nazionale delle Ricerche (IAS—CNR), Campobello di Mazara, 91021 Trapani, Italy; fabio.dagostino@cnr.it; 4 Dipartimento di Riabilitazione, Fragilità e Continuità delle Cure, Unità di Medicina Orale, Policlinico Universitario Palermo, 90127 Palermo, Italy; giuseppina.campisi@unipa.it

**Keywords:** cytisine, mucosal permeability, smoking cessation therapy, spray-drying, buccal tablets

## Abstract

Cytisine (CYT) has emerged as a promising molecule to treat nicotine addiction, since it acts as a partial agonist of nicotinic acetylcholine receptors. However, its unfavorable pharmacokinetic properties lead to multiple administrations per day, reducing the patient’s compliance and increasing the side effects. To overcome these drawbacks, CYT buccal administration is here proposed. Firstly, CYT stability in the buccal environment was assessed and its intrinsic ability to permeate/penetrate the tissue was determined by applying CYT solutions at increasing concentrations. Furthermore, a spray-drying method was selected and optimized as it is an eco-friendly, easily scalable and effective technique to obtain uniform and reproducible CYT-loaded (5% *w*/*w*) pharmaceutical powders, which were directly compressed, thus obtaining different buccal delivery systems (BDSs). The obtained BDSs were homogeneous and reproducible and embedded CYT in its amorphous form. The mechanism of CYT release was evaluated in vitro and found to be mainly driven by a Fickian diffusion phenomenon. Predominantly, the ex vivo permeation assays highlighted the ability of the BDSs to enhance CYT permeation, also producing high drug fluxes through the mucosa. Speculative mathematical evaluations based on the already-known CYT pharmacokinetic parameters showed that CYT-loaded BDSs could potentially be sufficient to obtain a therapeutic effect, thus making the reported formulations suitable candidates for further in vivo trials.

## 1. Introduction

Currently, cigarette smoking is a serious public health problem, since it is the primary worldwide cause of death and morbidity. Chronic exposure to nicotine and other substances from tobacco combustion causes several diseases, such as lung cancer and heart stroke [1]. Despite the fact that smoking cessation strongly reduces premature mortality while increasing the quality of life, it is extremely challenging for smokers to quit because of nicotine psychopharmacological effects leading to addiction [2,3]. To prevent addiction behavior, some strategies based on nicotine administration (known as nicotine replacement therapies; NRTs) have recently been proposed, by administering various dosage forms such as gum, transdermal patch, tablet, and nasal spray. All of these cause nicotine release over a period of minutes (e.g., nasal spray and tablet) or hours (e.g., transdermal patches), thus reducing the craving and alleviating tobacco withdrawal symptoms [4]. Even though NRTs are proven to increase the chances of smoke quitting by 70%, they still contain nicotine that could exacerbate a pre-existing toxicity condition or cause chronic damage over time [5]. Other first-line therapeutic options are bupropion and varenicline. Bupropion is an antidepressant able to inhibit the neuronal reuptake of dopamine and noradrenaline. Its mechanism of action against tobacco addiction is not fully understood, although it seems to confer both anti-craving and anti-withdrawal effects by inhibiting dopamine’s reuptake. In 1997, the FDA approved its therapeutic use for tobacco cessation treatment and thus a sustained-release dosage form is still marketed. Similarly, to other antidepressants, the most common side effects are insomnia, headache, dizziness, weight loss, dry mouth, nausea and vomiting. Varenicline is also a nicotinic acetylcholine receptors (nAChRs) partial agonist, selective for the α4β2 form which is related to dopamine release due to nicotine binding. Thus, varenicline is able to decrease the intensity of withdrawal symptoms and rewarding effects, while reducing the perceived pleasure generated by nicotine consumption, helping smokers to achieve and maintain tobacco abstinence. However, common side effects are nausea, insomnia, abnormal dreams, headache, nasopharyngitis, and xerostomia [6]. For this reason, research is now focused on the identification of new substances or alternative strategies to successfully achieve smoking cessation. In particular, in recent years, cytisine (CYT) has gained considerable and increasing interest in the scientific community. CYT is a plant-based alkaloid obtained from Cytisus laburnum seeds and acts as a partial agonist of nicotinic acetylcholine receptors (nAChRs), thus being a potential anti-smoking substance [7]. CYT has been proved to be as effective as varenicline while also being cheaper, causing fewer side effects and improving the patients’ compliance. Furthermore, its efficacy in prolonging the period of abstinence from smoking compared to placebo and NRTs treatment has also been demonstrated [8,9,10]. Currently, two available CYT-loaded products, Tabex^®^ and Desmoxan^®^, (CYT dose: 1.5 mg) in the form of tablets/capsules for oral administration have been already approved in western and central Europe as medicines for smoking cessation. However, CYT is characterized by unfavorable pharmacokinetic properties including short half-life (4.4 ± 0.5 h) and high apparent volume of distribution (110.1 ± 19.0 L), which lead to the need for multiple administrations per day, thus reducing the adherence to therapy [9]. Indeed, the approved therapeutic regimen consists of a 25-day treatment, starting from 6 tablets/capsules per day, which are slowly decreased to 1–2 doses daily. Furthermore, this therapeutic strategy does not allow a steady state to be reached, and this leads to the need for doses at close intervals (every 2 h at the beginning of the therapy) and on-off drug intake (e.g., when taking six doses: one every 2 h, followed by no administration for 12 h) [11]. Considering the potential of CYT in smoking cessation, together with the disadvantages due to oral administration, a valuable and innovative approach to maximize the use of this promising molecule, could be represented by its delivery through the buccal mucosa. Buccal drug delivery offers an easily accessible and non-invasive route of administration and allows direct absorption of substances into the systemic circulation, thus bypassing the first pass effect and increasing the overall bioavailability [12]. An ideal buccal delivery system (BDS) should be solid, to be easily administrable. and able to assure a sustained and controlled drug release profile, thus minimizing the need for multiple administration. Furthermore, it might also act as an effective absorption enhancer system. There are several methods of preparing BDSs, such as solvent casting, electrospinning, and direct compression of spray-dried or freeze-dried powders. Recently, spray-drying has become an attractive technique in food and pharmaceutical fields, due to the benefit of obtaining submicron-to-micron scale powders having homogeneous particle distribution, size, and shape, together with the high versatility and scalability of the process [13]. To summarize, the key points of the present work are the following: (i) CYT has recently emerged as a promising useful anti-smoking agent which has already proved to be as effective as varenicline, while being cheaper and causing fewer side effects; (ii) CYT is already marketed in western and central Europe as medicines for smoking cessation in the form of tablets/capsules for oral administration; however, due to CYT’s unfavorable pharmacokinetic properties, the therapeutic regimen required is very close and complicated, leading to fluctuation of plasma concentration and low patient compliance; (iii) buccal drug delivery could be an attractive alternative to overcome the existing limits of the oral route, due to direct absorption into the systemic circulation; (iv) the spray-drying method could be an eco-friendly, easily scalable and effective technique to obtain CYT loaded pharmaceutical powders to be directly compressed in CYT-loaded buccal tablets. In view of these considerations, this work firstly aims to assess the intrinsic ability of CYT to permeate throughout the buccal mucosa (evaluation of the permeability coefficient Kp), as this could give a starting idea of the possibility to benefit from the buccal route for CYT administration. Subsequently, a further aim was to design, develop, and characterize novel CYT-loaded BDSs obtained by direct compression of CYT-loaded spray-dried polymeric matrices. For this aim, different spray- dried powders were prepared by varying the excipient composition and polymer/additive ratios. The resulting powders and tablets were evaluated in terms of homogeneity, reproducibility, CYT physical state and drug release behavior. Mainly, the ability of the proposed BDSs to act as CYT permeation enhancers was demonstrated by carrying out ex vivo experiments. Additionally, some mathematical correlations between the experimental results and per os CYT already-known pharmacokinetics parameters are reported. Considering the experimental drug fluxes through the buccal mucosa, the reported BDSs could represent a promising and effective alternative strategy to overcome the limitations of the currently available CYT oral smoking cessation therapy.

## 2. Materials and Methods

### 2.1. Materials

Cytisine (CYT) was kindly supplied from A.C.E.F. Spa (Fiorenzuola D’Arda, Italy). Eudragit^®^ RS100 was obtained from Rofarma (Milan, Italy). Polyvinylpyrrolidone K90 (PVP K90), propylene glycol, xylitol, and polyethylene glycol 1000 (PEG 1000) were purchased from Farmalabor (Canosa di Puglia, Italy). Trifluoroacetic acid (TFA) was obtained from Merck (Darmstadt, Germany). The isotonic saline solution (0.9% *w*/*v*) was prepared by dissolving 9 g of sodium chloride (NaCl) in 1 L of distilled water. The isotonic saline solution containing trehalose (5% *w*/*v*) was prepared by dissolving 9 g of NaCl and 50 g of trehalose in 1 L of distilled water. Simulated salivary fluid (pH 6.8) was prepared by dissolving NaCl (0.126 g), KCl (0.937 g), NaHCO_3_ (0.631 g), KSCN (0.189 g), KH_2_PO_4_ (0.655 g), urea (0.200 g), Na_2_SO_4_ (0.154 g), NH_4_Cl (0.178 g), and CaCl_2_ (0.130 g) in 1 L of distilled water [14]. Phosphate buffered saline (PBS) (pH 7.4) was prepared by solubilizing NaCl (8.00 g), KCl (0.20 g), KH_2_PO_4_ (0.24 g), and Na_2_HPO_4_ ∙ 2 H_2_O (1.81 g) in 1 L of distilled water [15].

All chemicals and solvents (analytical grade) were purchased from Carlo Erba Reagents (Milan, Italy) and were used without further purification. Porcine buccal tissues were kindly supplied by the Municipal Slaughterhouse of Villabate (Palermo, Italy).

### 2.2. Pre-Formulation Studies of Cytisine (CYT)

#### 2.2.1. Analytical Methods to Quantify CYT

- UV-Vis Methods

UV-VIS analyses were performed using a Shimadzu 1601 Instrument (Kyoto, Japan). CYT concentration in different dissolution media was measured using the appropriate calibration curves and blanks with simple, accurate and reproducible methods.

Two calibration curves in phosphate buffer (pH 7.4) were obtained at λ_max_ = 305 nm; linearity range 0.0006–0.0240 mg/mL, regression curve Abs = 0.0052 + 39.13 ∙ C_CYT_ in [mg/mL] (where C_CYT_ is the cytisine concentration in [mg/mL]) (R= 0.999; SE= 0.009).

Two calibration curves in methanol were obtained at λ_max_ = 309 nm: linearity range 0.0005–0.0080 mg/mL, regression curve Abs = 0.04342 + 41.15 ∙ C_CYT_ in [mg/mL] (R = 0.999 SE = 0.005); linearity range 0.006–0.020 mg/mL, regression curve Abs= 0.02025 + 39.08 ∙ C_CYT_ in [mg/mL] (R = 0.999; SE = 0.005).

No interference between CYT and the other components of the formulations was observed at the testing concentrations, and no changes in drug absorbance at its λ_max_ were experienced in the presence of the excipients. Analogously, the amount of drug recovered in the acceptor chamber and/or entrapped into the mucosal membrane was measured after withdrawal or extraction from mucosal tissue by methanol. Intraday and interday variations observed during the collection of the experimental data were lower than sensibility.

- HPLC Method

HPLC analyses were performed using a HPLC Shimadzu LC-10AD VP Instrument (Tokyo, Japan) equipped with a binary pump LC-10AD VP, a UV SPD-M20A Diode Array Detector, a 20 μL loop injector and a computer integrating apparatus (EZ Start 7.4 software, Shimadzu Scientific Instruments, Inc., Columbia, MD, USA). Chromatographic separation was achieved via a reversed-phase column ACE^®^ EXCEL 5 CN-ES (5 μm, 4.6 × 125 mm; Advanced Chromatography Technologies Ltd., Aberdeen, UK) as a stationary phase, while acetonitrile and TFA 0.1% (*v*/*v*) in Milli-Q Water (10:90 *v*/*v*) were used as a mobile phase in isocratic conditions. The flow rate was set at 1 mL/min and the UV-VIS wavelength at 305 nm (observed range 200–700 nm). In these conditions, CYT retention time was 2 min. A calibration curve was used for quantification of peaks. The calibration curve was performed in the concentration range of 0.004–0.100 mg/mL, injecting standard solutions of CYT in Milli-Q water. The regression curve was Area = 5.26∙10^4^ + 5.11∙10^7^ ∙ C_CYT_ in [mg/mL]. HPLC reports were highly reproducible and linearly related to concentration (R = 0.999).

#### 2.2.2. CYT Stability Assay

CYT standard solutions were prepared by dissolving a carefully weighed amount of CYT either in PBS (pH 7.4) or simulated salivary fluid (pH 6.8; initial concentration: 0.01 mg/mL). Each solution (10 mL) was kept at 37 ± 0.5 °C under continuous stirring (Heidolph MR3001K Hotplate Stirrer with Heidolph EXT3001 Temperature Probe, Heidolph Instruments, Schwabach, Germany) for 6 h. Every 30 min, samples (500 µL) were withdrawn and analyzed to determine CYT concentration by both UV-VIS and HPLC-DAD analyses, as reported above. Each experiment was performed in triplicate. Data are reported as percentage variation of CYT concentration over time (means ± SE) compared to the starting standard CYT concentration value (100%).

#### 2.2.3. Ex Vivo Permeation/Penetration of CYT through Porcine Buccal Mucosa

- Tissue preparation

Porcine buccal mucosae were obtained from tissue removed from the vestibular area of the retromolar trigone of freshly slaughtered domestic, 6–8 month-old, pigs (intended for human consumption, therefore their use does not require ethical approval). The animal specimens were collected immediately after animal slaughter and transferred within 1 h to the laboratory in a refrigerated transport box. Here, they were washed in isotonic saline solution and excised to remove any excess of tissue. Samples were then placed in a trehalose-containing isotonic solution (5% *w*/*v*), left for 1 h and subsequently kept at −80 °C for at least one week. Before the ex vivo permeation studies, the tissue samples were equilibrated at room temperature, washed for 1 h in isotonic solution and then subjected to thermal shock to obtain the buccal mucosa specimens. Briefly, samples were dipped for approximately 1 min in a pre-warmed isotonic solution (60.0 ± 0.5 °C) and then the mucosa was carefully peeled off from the adipose tissue and connective tissue manually, to obtain the heat-separated epithelium (slides 250 ± 25 μm thick, evaluated by a digital micrometer VWR International, Milan, Italy), along with the intact basal lamina [16,17].

- Ex vivo permeation assay

To evaluate CYT permeation through porcine buccal mucosa, vertical Franz-type diffusion cells (Permeagear, flat flange joint, 9-mm orifice diameter, 15 mL acceptor volume, SES GmbH—Analysesysteme, Bechenheim, Germany) were used as a two-compartment open model. To remove all the biological matter that could interfere with the following drug quantification analysis, the previously obtained buccal mucosa was equilibrated in isotonic solution overnight at room temperature. Franz cells were mounted using adequate sections of the mucosa as a membrane between the acceptor and the donor chambers, filled with PBS (pH 7.4) and simulated salivary fluid (pH 6.8) and left to equilibrate at 37.0 ± 0.5 °C for 15 min. Hereafter, the donor fluid was removed and replaced with 0.5 mL of CYT solutions in simulated salivary fluid (pH 6.8) at different concentrations (2, 5, 8, 10 and 15 mg/mL). At scheduled time intervals (every 30 min), samples (0.5 mL) were withdrawn from the acceptor compartment and immediately replaced with fresh acceptor fluid to maintain the sink conditions. The amount of permeated drug was quantified spectrophotometrically using the relative calibration curve as described above. Each experiment was carried out for 6 h at 37.0 ± 0.5 °C under continuous magnetic stirring and repeated 6 times. Results are expressed as mean ± SE.

- Evaluation of CYT amount entrapped into the buccal tissue

At the end of each permeation experiment, the Franz cells were disassembled and the porcine mucosa was collected to evaluate the amount of CYT entrapped into the tissue. The collected buccal tissue was firstly washed with distilled water (2 mL) to remove any drug residue on the surface and then dipped for 2 min in 2 mL of warm methanol (55 ± 5 °C) to extract the entrapped CYT. The extraction procedure was repeated twice. The extraction liquors were then collected in a 10 mL flask and brought to volume with methanol. The amount of drug was quantified spectrophotometrically using the relative calibration curve, as described above. Results are reported as means ± SE.

#### 2.2.4. Determination of the Biopharmaceutical Parameters: Js, Kp, t_lag_, De and Ac

Drug flux (Js) through the buccal mucosa was calculated at the steady state per unit area by linear regression analysis of permeation data according to the following equation:

(1) $$\mathrm{Js}=\frac{Q}{A\cdot \;t}\;(µg/{\mathrm{cm}}^{2}\;\cdot \;h^{-1})$$

where Q is the amount of CYT (µg) permeated during the time interval t (h) and A is the area of the buccal mucosa available for permeation (0.636 cm^2^). At the steady state, Js is equal to the slope of the obtained straight line. The constant of permeability (Kp) was then calculated by the relationship:

(2) $$\mathrm{Kp}=\frac{\mathrm{Js}}{\mathrm{Cd}}\;(\mathrm{cm}/h)$$

where Cd is the initial CYT concentration loaded into the donor compartment.

The t_lag_ (min) was determined from the interception with the x axis of the obtained straight line at the steady state.

Similarly, at the end of each experiment, the amount of drug entrapped (De) per unit area into the mucosal tissue was calculated using the following relationship:

(3) $$\mathrm{De}=\frac{Q_{T}}{A}\;(µg/{\mathrm{cm}}^{2})$$

where Q_T_ is the amount (µg) of CYT entrapped into the tissue and A is the area available for accumulation (0.636 cm^2^). Accordingly, the accumulation (Ac) parameter was then calculated as follows:

(4) $$\mathrm{Ac}=\frac{\mathrm{De}}{\mathrm{Cd}}\;(\mathrm{cm})$$

where Cd is the starting CYT concentration loaded into the donor compartment [18,19].

Origin 8.5 software was used for mathematical data processing and results are expressed as means ± SE.

### 2.3. Preparation and Characterization of CYT-Loaded Buccal Tablets

#### 2.3.1. Preparation of CYT-Loaded Pharmaceutical Matrices by a Spray-Drying Technique

To prepare the CYT-loaded pharmaceutical matrices a spray-drying technique was used. Five percent (*w*/*v*) solutions (referring to the whole components) in methanol, ethanol or acetonitrile were prepared, according to the different compositions reported in Table 1. Specifically, 1 g of excipients together with CYT in the appropriate ratios were dissolved one by one in 20 mL of the chosen solvent. Eudragit^®^ RS 100, PEG 1000, propylene glycol (when present), xylitol (when present), PVP K90 (when present), and finally CYT were added in this order under magnetic stirring until a clear and homogeneous solution was formed. The clear solutions obtained by using both methanol and ethanol were processed to obtain CYT-loaded pharmaceutical powders using a BUCHI MINI Spray Dryer B-290 equipped with an inert loop B-295, set to the following parameters: inlet temperature 110 °C (ethanol solutions) or 75 °C (methanol solutions); solution flow 100 mL/h; nitrogen aspiration 100%; cooling temperature (inert loop) −20 °C. Firstly, the instrument was equilibrated with 20 mL of fresh solvent, and subsequently the prepared samples were processed. Afterwards, the instrument was cleaned with an additional 20 mL of fresh solvent. Finally, the obtained powders were recovered. Each powder batch (in terms of composition) was prepared in triplicate.

#### 2.3.2. Yield and Uniformity of CYT-Loaded Spray-Dried Powdery Matrices

The suitability of the spray-drying process was determined by evaluating the yield of the procedure as well as powder uniformity in terms of drug content. Firstly, the recovered spray-dried powders were carefully weighed using an analytical five decimal balance (mod. AE 240, Mettler-Toledo S.p.A., Milan, Italy), to calculate the yield of the spray-drying process as follows:

(5) $$\mathrm{Yield}\;\%=\frac{\mathrm{recovered}\;\mathrm{powder}\;\left(g\right)}{\mathrm{starting}\;\mathrm{components}\;\mathrm{amount}\;\left(g\right)}\times 100$$

Afterwards, randomly selected powder aliquots were accurately weighed (5 mg), transferred in a 10 mL flask, dissolved in methanol, and brought to volume with the same solvent. The resulting clear solutions were properly diluted and immediately subjected to UV-Vis as well as HPLC-DAD analyses to calculate the CYT amount and evaluate powder homogeneity. The drug loading percentage (DL%) and the loading efficacy percentage (LE%) values were then calculated as follows:

(6) $$\mathrm{DL}\%=\frac{\mathrm{CYT}\;\left(\mathrm{mg}\right)}{\mathrm{powder}\;\mathrm{amount}\;\left(\mathrm{mg}\right)}\times 100\;$$

(7) $$\mathrm{LE}\%=\frac{\mathrm{recovered}\;\mathrm{CYT}\;\mathrm{amount}\;\left(\mathrm{mg}\right)}{\mathrm{theorethical}\;\mathrm{CYT}\;\mathrm{amount}\;\left(\mathrm{mg}\right)}\times 100\;$$

Each experiment was performed in triplicate and data are reported as means ± SE. The prepared powders were considered uniform when low standard error values were obtained.

#### 2.3.3. Preparation of CYT-Loaded Buccal Tablets

CYT-loaded buccal tablets were immediately prepared by compressing the previously freshly prepared spray-dried powders. Carefully weighed amounts of powder (20 mg) were directly compressed (10 tons) by using a hydraulic, single-die tableting machine (Perkin Elmer IR Accessory, Waltham, MA, USA), two flat-faced punches and a die [20]. Tablets of 0.65 cm of diameter were obtained.

#### 2.3.4. Reproducibility of CYT-Loaded Tablets

To assess the reproducibility of the preparation method employed to obtain the CYT-loaded buccal tablets, weight (analytical balance, Mettler Toledo AE240, Columbus, OH, USA) and thickness (Digital Micrometer DIN 863IP40, ABS-system, 0–25 mm/0–1 inch, Vogel Germany, Kevelaer, Germany) were measured. Data are reported as means ± SE. Batches were considered uniform when standard error values within the ±10% range were obtained. Each experiment was performed on three randomly selected tablets for each batch of powder.

#### 2.3.5. Fourier Transform Infrared Spectroscopy (FTIR) in Attenuated Total Reflectance (ATR) Mode Analysis

FTIR-ATR mode spectra were recorded using a Fourier Transform Infrared Spectrometer (Nicoret iS5, Thermo Scientific™, Waltham, MA, USA) equipped with a ZnSe ATR unit ID7 (Thermo Scientific™, USA) for surface analysis. Spectra were collected by the accumulation of 32 scans in the 4000–500 cm^−1^ spectral range with a resolution pair to 2 cm^−1^, and rationed to the appropriate background spectra. The following samples were analyzed: pure CYT, Eudragit^®^ RS100 and CYT physical mixture, blank spray-dried powders composed only of Eudragit^®^ RS100 and CYT, and CYT-loaded spray-dried powders.

#### 2.3.6. X-ray Diffraction (XRD) Analysis

The drug dispersion state in CYT-loaded BDS was assessed by an X-ray diffractometer suitable for powders (D-8 Focus, Bruker, Billerica, MA, USA). The diffractograms were obtained from 5° to 60° in ⊖/2⊖ at 2°/min, 40 kV voltage and 30 mA current at room temperature. XRD analyses were performed on pure CYT and CYT-loaded powders immediately after their preparation, as well as after one month of sample storage (at room temperature).

#### 2.3.7. In Vitro Dissolution Studies and Drug-Release Kinetics Evaluation

The ability of the prepared BDSs to release CYT was evaluated by in vitro dissolution studies using the flow through system previously described [21]. Briefly, the system is composed of a container filled with simulated salivary fluid (pH 6.8; 100 mL) from which the liquid is forced at a constant rate (0.3 mL/min) by a peristaltic pump (uniPERISTALTICPUMP 1, LLG-Labware, Meckenheim, Germany) to a release chamber in which the prepared tablets were located. In these conditions, the salivary film wetting the formulations was about 0.1 mm thick. Each experiment was performed at 37 ± 0.1 °C by placing a beaker with the simulated salivary fluid in a thermostatic bath. At scheduled time intervals (every 5 min), aliquots (1.5 mL) of solution from the release chamber were collected and the drug amount was determined spectrophotometrically, using the appropriate blank and calibration curve, as reported above. The amount of drug released and the residual drug content in the tablet matched the drug content value calculated by the DL%. The experiment was carried out for 2.5 h and performed in quadruplicate. To evaluate CYT release behavior, dissolution data were measured using Origin 8.5 and fitted to the semi-empirical equations usually applied to evaluate kinetics of drug release from delivery systems [22].

#### 2.3.8. Ex Vivo Permeation/Penetration of CYT through Porcine Buccal Mucosa by Administering CYT-Loaded BDSs

The ex vivo permeation/penetration studies by administering CYT-loaded BDSs were carried out in accordance with those described in Section 2.2.3. Each prepared CYT-loaded BDS was loaded into the donor compartment of the vertical Franz-type diffusion cell and soaked with 500 µL of simulated salivary fluid (pH 6.8), while 15 mL of PBS (pH 7.4) was used as acceptor fluid. Each experiment was repeated six times and performed at 37 ± 0.5 °C for 6 h. Aliquots (0.5 mL) of acceptor fluid were withdrawn at scheduled time intervals, replaced with fresh medium, and analyzed as already reported. At the end of each permeation experiment the donor medium was withdrawn, centrifuged, appropriately diluted in methanol, and spectrophotometrically analyzed (as described above) to determine the actual CYT concentration in the donor chamber to be employed as the correct Cd value to calculate the Kp and Ac parameters. Moreover, at the end of the experiments each tablet was subjected to visual and tactile inspections to verify its integrity. Finally, the amount of CYT entrapped into the buccal tissue was extracted by methanol and quantified as described above. Data were further examined to calculate the previously described biopharmaceutical parameters. Results are expressed as means ± standard error.

### 2.4. Data Analysis

Data are expressed as mean ± standard error (SE). All differences were statistically evaluated by one-way analysis of variance (ANOVA or F-test) with the minimum level of significance set at *p* < 0.05.

## 3. Results

### 3.1. Pre-Formulation Studies of CYT

#### 3.1.1. CYT Stability in PBS (pH 7.4) and Simulated Saliva (pH 6.8)

**Figure 1:**
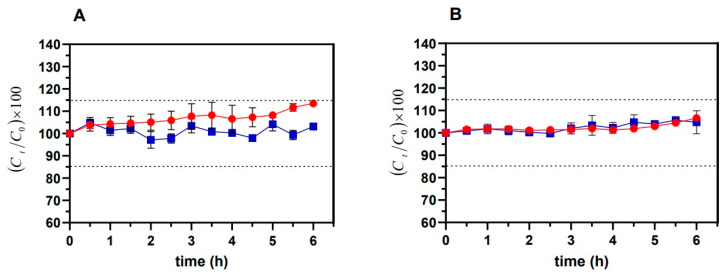
Percentage variation of the starting CYT concentration (100%) over time in (**A**) simulated salivary fluid (pH 6.8) and (**B**) PBS (pH 7.4). The measurements were carried out by UV-Vis (red) and HPLC-DAD (blue) methods. Results are presented as means ± SE (n = 3).

CYT concentration in PBS and simulate saliva (Figure 1A,B, respectively) was monitored over time by both UV-Vis and HPLC-DAD methods. As shown in Figure 1, by setting the CYT starting amount at 100%, only small fluctuations (≤±15%) were observed for the whole duration of the experiments in both the tested media. Moreover, neither changes in terms of the CYT UV-Vis spectrum (shape) and CYT chromatogram (peak shape, retention time) nor novel degradation peaks were observed.

#### 3.1.2. CYT Permeation through the Buccal Mucosa and Tissue Accumulation

**Figure 2:**
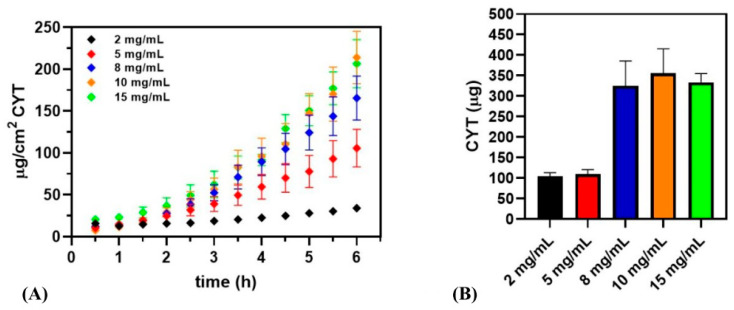
Permeation/penetration results by administering solutions at the following concentrations: 2 mg/mL (black), 5 mg/mL (red), 8 mg/mL (blue), 10 mg/mL (orange) and 15 mg/mL (green). (**A**) CYT (µg/cm^2^) permeated as a function of incubation time (h) and (**B**) CYT (µg) accumulated into the buccal membrane after 6 h of the experiment. Data are presented as mean ± SE (n = 6).

The aptitude of CYT to permeate or penetrate the buccal membrane was evaluated by ex vivo experiments testing CYT solutions at different concentrations. Figure 2 reports both drug permeation profiles (Figure 2A) and the amount of CYT accumulated into the buccal tissue after 6 h (Figure 2B). It is notable that the amount of CYT permeated increases with rising CYT concentration into the donor chamber until tissue saturation occurs, in the experimental conditions used. Indeed, the results of the two highest concentrations tested (10 mg/mL and 15 mg/mL) nearly overlapped. It is also observable that CYT in the acceptor compartment is already quantifiable at the first considered time point (30 min), indicating the good aptitude of CYT to cross the buccal mucosa. As a consequence, in calculating the biopharmaceutical parameters (Table 2), the extrapolated t_lag_ indicates the time interval needed to reach the flux equilibrium (steady state conditions).

Lastly, the extrapolated biopharmaceutical parameters are reported in Table 2. In particular, Js, De and Ac values are in accordance with data reported in Figure 2. However, to better evaluate CYT permeability, the Kp (permeability constant) value must be considered. The Kp should be constant for each molecule as it does not depend on drug concentration. Nevertheless, it could depend on the experimental conditions: when administering 2 mg/mL solution, the CYT amount that results is too low to observe its permeability, whereas the maximum concentration tested (15 mg/mL) is probably too high inasmuch as membrane saturation has occurred, leading to misleading results. Consequently, the effective Kp value for CYT is the mean of the Kp values obtained administering 5, 8 and 10 mg/mL solutions, respectively, resulting in 0.00447 ± 0.00027 cm/h. The amount of CYT entrapped into the buccal tissue further confirms the above statement. Indeed, as reported, the entrapment data at the end of the ex vivo experiments for the 8, 10 and 15 mg/mL solutions overlap. In particular, the maximum amount of CYT accumulated into the buccal tissue (tissue saturation) was ≈ 500 µg/cm^2^. Moreover, the required time (t_lag_) to establish the steady state equilibrium increases with increasing the CYT concentration into the donor chamber.

### 3.2. Design, Preparation, and Characterization of CYT-Loaded Buccal Tablets

#### 3.2.1. Spray-Dried Matrix Powders, Tablet Preparation and Preliminary Characterization

Four different compositions (Table 1, Materials and Methods section) were tested to prepare the CYT-loaded pharmaceutical powders using a spray-drying technique. Furthermore, three different solvents (methanol, ethanol and acetonitrile) were tested, according to the literature regarding the spray-drying process of Eudragit^®^ RS100, to evaluate their effects on the suitability of the whole procedure [23]. However, acetonitrile was immediately discarded due to xylitol lack of solubility in it. This decision was based on the choice to employ the same solvent for all the formulations, and thus the xylitol-free composition was also not subjected to a spray-drying process by preparing an acetonitrile solution. Ethanol and methanol were able to easily and rapidly dissolve all the selected components (excipients and drug), and thus were both used. However, the changes in terms of both the solvent employed and the inlet temperature consequently required, allowed us to obtain some different powders. In particular, powders obtained by ethanol solutions resulted in large grains, often compacted to form hard sheets which were difficult to recover and unpack into finer grains. Consequently, ethanol was discarded as the obtained powders were difficult to handle. In contrast, the powders obtained by spray-drying methanol solutions were fine, easy to recover and generally very convenient. Thus, the reported data below are those related to the pharmaceutical matrix powders obtained from methanol solutions. After optimization of the spray-drying process, all the obtained powders showed a white color and the appearance of small grains/flakes. The suitability of the procedure was evaluated in terms of yield as well as drug loading. Data are reported in Table 3 and highlight the reproducibility of the preparation technique as well as sample homogeneity and desirable yield values.

The freshly obtained pharmaceutical powders were directly compressed to obtain CYT-loaded buccal tablets, subsequently characterized in terms of drug content, thickness and weight variation, as reported in Table 3. All data suggest high product reproducibility and homogeneity.

#### 3.2.2. FTIR in ATR Mode and XRD Analyses

**Figure 3:**
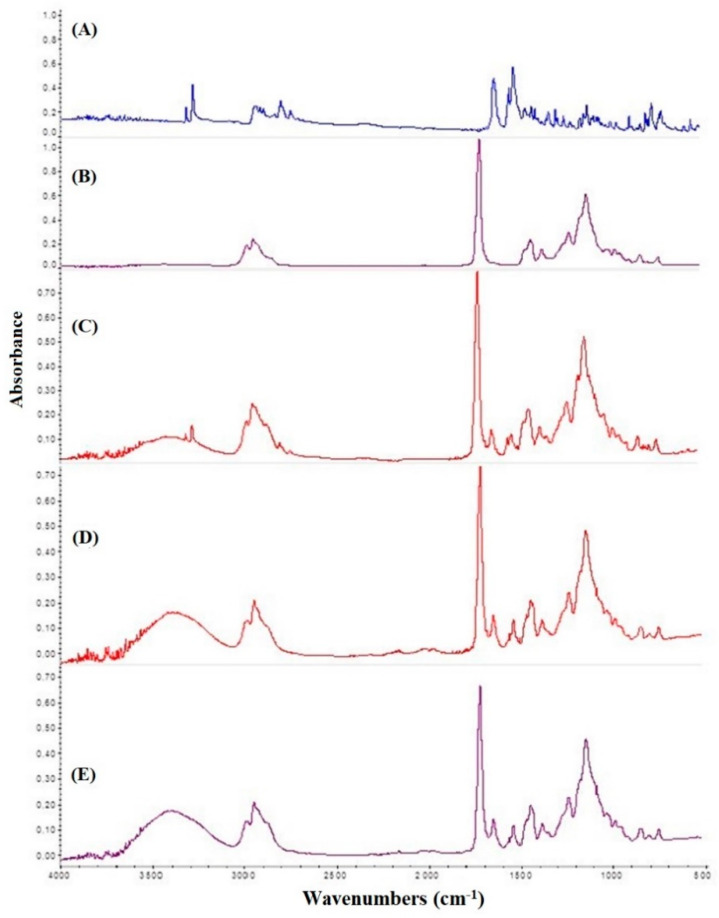
FTIR in ATR mode spectra of (**A**) pure CYT; (**B**) Eudragit^®^ RS100; (**C**) Eudragit+CYT physical mixture; (**D**) Eudragit+CYT blank spray-dried sample; (**E**) S-CYT-B spray-dried powder (reported as a representative example for all the prepared powders).

FITR analyses were performed in order to investigate any drug–excipient chemical interactions. The spectrum obtained for pure CYT (Figure 3A) shows the following characteristic bands: 1560 cm^−1^ (C=H aromatic bonding), 1642 cm^−1^ (C=O amide stretching), 3278 and 3319 cm^−1^ (N-H stretching of secondary amine) and the 2745–2930 cm^−1^ range (C-H stretching). The Eudragit^®^ RS100 FTIR spectrum (Figure 3B) is characterized by the following bands: 1750 cm^−1^ (C=O ester stretching) and 2530–3000 cm^−1^ range (N-H broad amine salt stretching). The FTIR spectrum of the Eudragit^®^ RS100-CYT physical mixture (Figure 3C) results from an overlapping of the pure substances’ spectra. However, when the latter compounds were subjected to spray-drying, the resulting FTIR data were characterized by the disappearance of CYT bands at 3278 and 3319 cm^−1^, as observable both in the blank sample (Eudragit^®^ RS100+CYT powder obtained by spray-drying) and in the prepared pharmaceutical powders (S-CYT-B is reported here as a representative sample), as shown in Figure 3D,E respectively. This phenomenon is probably due to the overlapping of the OH band of the hygroscopicity of the matrix. Indeed, the OH band shown in Figure 3C,D from 3700 to 3050 cm^−1^ is more intense than in Figure 3A,B. Furthermore, regarding this, to exclude any alteration of the CYT caused by the spray-drying process, the pharmaceutical powders were analyzed by HPLC-DAD. Chromatograms revealed the same sharp peak at Rt of 2 min, having UV-Vis absorption spectrum of CYT with λ_max_ at 305 nm, and the absence of chromatographic peaks for an elution time over 20 min, showing no presence of degradation products. The quantitative determination of CYT also corresponded to that obtained by UV-Vis analyses. This evidence ruled out any doubt about the maintenance of CYT stability during the powder production process and suggested an interaction occurring between the drug and the main polymer caused by the spray-drying process.

**Figure 4:**
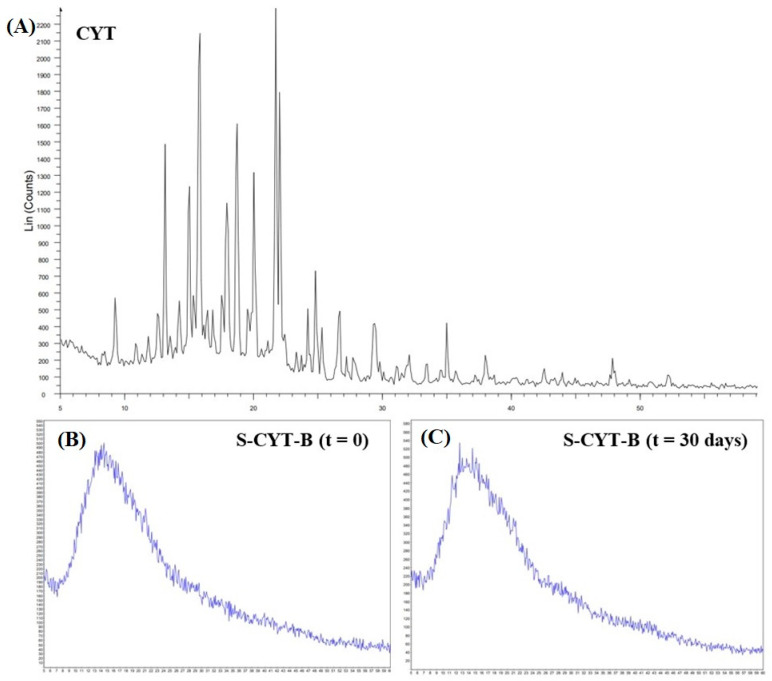
X-ray diffraction patterns of (**A**) pure CYT, (**B**) S-CYT-B powder immediately after preparation and (**C**) S-CYT-B powder after 30 days storage. S-CYT-B spray-dried powder was reported as a representative example for all the prepared powders.

The XRD spectra both of pure CYT and of all matrix powders were recorded. The diffractogram of pure CYT (Figure 4A) showed the characteristic peaks of crystalline arrangement. In contrast, the diffractogram of the spray-dried powder (Figure 4B; S-CYT-B is reported here as a representative sample) revealed the absence of the latter peaks, suggesting the occurrence of complete amorphization of both CYT and xylitol during the matrices preparation. It is noticeable that, after 30 days of storage at room temperature, the powders were still able to prevent CYT and xylitol re-crystallization, thus remaining stable (Figure 4C).

#### 3.2.3. CYT Release Studies

**Figure 5:**
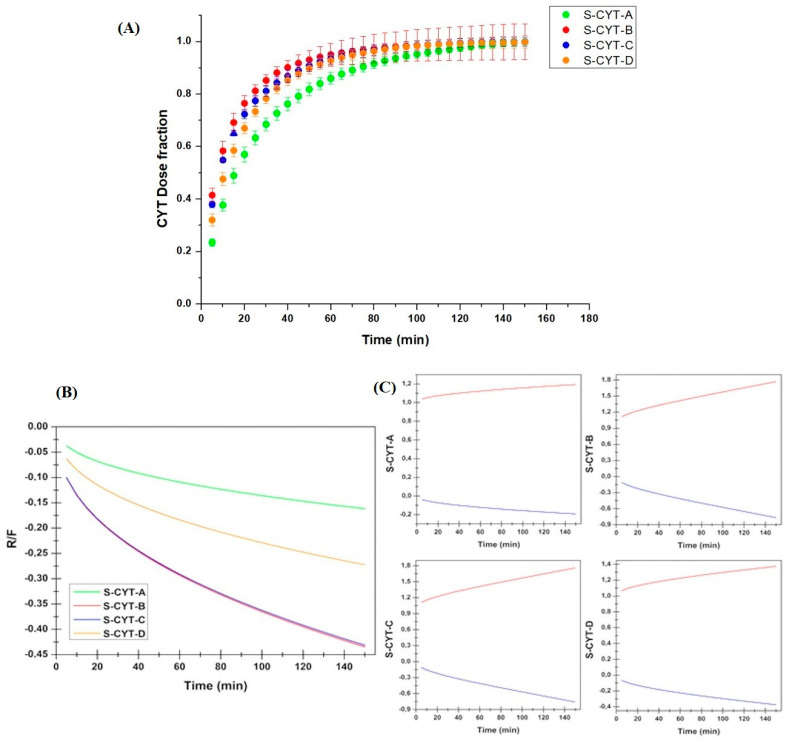
(**A**) Dose fraction of CYT released as a function of time (h) from S-CYT-A (green), S-CYT-B (red), S-CYT-C (blue) and S-CYT-D (orange) buccal tablets. Values are presented as means SE ± (n = 4) and evaluation of the Fickian diffusional contribution (F) and the Case II relaxation/erosional contribution (R) in the Peppas–Salhin model: (**B**) variation of the R/F ratio as a function of time; (**C**) detailed F (red) and R (blue) patterns for each formulation.

Similar discharge behaviors were observed for the S-CYT-B, S-CYT-C and S-CYT-D formulations, while S-CYT-A exhibited a different release trend (Figure 5A). The main changes between the discharge kinetics for the prepared tablets are mainly related to the release rate, rather than real kinetic differences. Moreover, all the proposed formulations are able to release the total amount of loaded CYT at the end of the in vitro dissolution tests. At the end of the experiments, all tablets recovered from the donor compartment were visually unchanged in shape.

To better understand the processes involved in the release phenomenon, data were curve fitted by considering several mathematical models. The calculated fitting parameters are reported in Table 4. As can be seen, the best results in terms of square of coefficient of determination were achieved by considering the Korsmeyer–Peppas (power law) and Peppas–Sahlin kinetics, although it is noticeable that anomalous experimental n values were obtained when curve fitting the power law equation.

To better understand the driving force governing the drug discharge, the Fickian diffusional contribution (F) and the Case II relaxation/erosional contribution (R) parameters were calculated as follows [24]:

(8) $$F=\frac{1\;}{1+\frac{k_{2}}{k_{1}}t^{m}}$$

(9) $$\frac{R}{F}=\frac{k_{2}\;\cdot {\;t}^{m}}{k_{1}}$$

Figure 5B,C report the obtained data both in terms of R/F behavior as a function of time and as detailed pattern of the two contributions. As can be seen, the Fickian contribution results dominate those of R.

#### 3.2.4. Ex Vivo Permeation/Penetration of CYT through Porcine Buccal Mucosa by Administering CYT-Loaded BDSs

**Figure 6:**
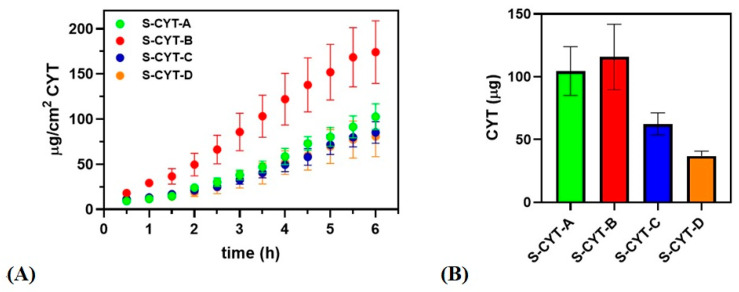
Permeation/penetration results of administering S-CYT-A (green), S-CYT-B (red), S-CYT-C (blue) and S-CYT-D (orange). (**A**) CYT (µg/cm^2^) permeated as a function of incubation time (h) and (**B**) CYT (µg) accumulated into the buccal membrane after 6 h. Results are presented as means ± SE (n = 6).

Figure 6A illustrates CYT permeation profiles as well as the amount of drug accumulated inside the porcine buccal mucosa after 6 h (Figure 6B) of administration of the proposed formulations in tablet form.

The experimental data were further examined to calculate the permeation/penetration parameters, as reported in Table 5. It is notable that all the proposed formulations are able to improve CYT permeability through the porcine buccal mucosa, when compared to CYT alone (Kp = 0.00447 cm/h), as confirmed by the significant increase of Kp. Generally, when CYT is embedded into buccal tablets its Kp value increases by an order of magnitude. In particular, even the worst formulation (S-CYT-D) allows at 2.8-fold enhancement of Kp (0.00447 vs. 0.01240 cm/h respectively), while a 7.6-fold increase (0.00447 vs. 0.03417 cm/h respectively) is observable for the best one (S-CYT-B). As reported, all the proposed formulations lead to a similar experimental drug concentrations in the donor chamber (≈1 mg/mL), which is lower than the minimum concentration previously tested when evaluating CYT by itself. It should be emphasized that the total amount of CYT administered here by a buccal tablet is ≈ 1 mg in 500 µL of fluid loaded into the donor chamber to soak the formulation. Consequently, the theoretical maximum CYT concentration in the donor compartment could be ≈2 mg/mL. All of the buccal tablets allow enhanced drug flux. Specifically, S-CYT-A, S-CYT-C and S-CYT-D result in Js values comparable to CYT 5 mg/mL solution or slightly lower, while with the S-CYT-B tablet, the flux through the mucosa is slightly lower than that observed for CYT 8 mg/mL solution. Furthermore, the application of the buccal tablets reduces the t_lag_ required to establish the steady state equilibrium (e.g., 23 min for S-CYT-B). The nature of the prepared BDSs, which behave like inert matrices, is confirmed by the results of the visual and tactile inspection conducted at the end of the permeation assays. Indeed, all the tablets loaded into the donor chamber remain perfectly intact and unchanged after 6 h of experiment.

## 4. Discussion

### 4.1. Pre-Formulation Studies of CYT

The therapeutic application of CYT as an alternative treatment for smoking cessation and tobacco dependance has attracted wide interest from the scientific community [10]. However, the short plasma half-life of this compound after oral administration leads to a particularly complex and close therapeutic regimen which could cause poor patient adherence and compliance to the treatment [9]. A promising solution to overcome the existing limits might involve such alternative routes of administration as application onto the buccal mucosa.

In this context, the first piece of information needed is the solubility and the stability of the selected molecule in the proper aqueous fluids related to the chosen route of administration: simulated salivary fluid (pH 6.8) as model medium in which CYT will be released after buccal administration and PBS (pH 7.4) as a model medium for the bloodstream (target site after buccal permeation).

As CYT is a water-soluble molecule, solubility evaluation in physiological media was not necessary, and thus its stability was firstly evaluated. Samples collected from the stability assays were subjected to both UV-Vis and HPLC-DAD analyses in order to not only evaluate CYT concentration but further highlight any occurring change in the UV-Vis spectrum, as well as any variation occurring in shape and retention time of the CYT peak or the presence of degradation product peaks. As reported, tight concentration fluctuations were highlighted and no variations in UV-Vis and HPLC-DAD behaviors were detected, thus confirming the stability of CYT under the tested conditions.

Subsequently, as CYT has never been investigated and administered via the buccal mucosa before, it is crucial that it has its intrinsic aptitude to penetrate and/or permeate the buccal membrane. The penetration/permeation experiments were conducted by using vertical Franz type diffusion cells as a two-compartment open model and porcine buccal mucosa as a valid model to simulate human epithelium [25,26,27]. To better understand CYT behavior, solutions of increasing concentrations were tested and the biopharmaceutical parameters (Js, Kp, De, Ac and t_lag_) were calculated and evaluated. The obtained results highlighted CYT’s intrinsic ability to permeate through the porcine buccal mucosa and reach the acceptor compartment. This is confirmed by the Js values, which increase together with the concentration of the solution loaded into the donor chamber, as well as by carefully observing the permeation data. Indeed, it is observable that CYT is already detectable in the acceptor chamber at the first considered time point. Furthermore, it is relevant to emphasize that the term t_lag_ is here used in a slightly incorrect way. Generally, the lag time refers to the time needed for the drug to appear into the acceptor chamber. However, in the present work, the calculated and reported t_lag_ values indicate the time necessary to establish steady state conditions. Generally, the term steady state indicates an unvarying condition in a physical process. In this context, a steady state is maintained by constant intake of drug into the acceptor chamber due to permeation. The steady state flux (Js) is thus represented by the slope of the linear portion of the permeation curve obtained by plotting the amount of drug permeated per unit area as a function of time. From the Js data it is then possible to calculate the constant of permeability (Kp), which is one of the main parameters to be considered as it gives information about permeation behavior that does not depend on drug concentration. As the permeation phenomenon is the result of different partition processes, firstly between the donor compartment and the membrane and then between the latter and the acceptor chamber, to fully appreciate coherent values, the experimental conditions should be considered. As a consequence, the five tested concentrations do not give comparable results. In particular, the real Kp value to be considered should be the mean of those obtained when evaluating the 5, 8 and 10 mg/mL solutions, which gave comparable values net of the experimental error. When studying lower or higher concentrations, the Kp values calculated gave misleading results. Particularly, when administering a 2 mg/mL CYT solution, it is likely that the total amount of administered CYT is too low to establish an actual steady flow, thus making the constant of permeability appear lower. In contrast, the administration of a 15 mg/mL CYT solution seems to saturate the available penetration/permeation area, as demonstrated by the observed drug flux, which is comparable to that obtained for the 10 mg/mL solution, as well as by the apparent reduction of the calculated Kp.

Furthermore, this assumption is confirmed by the data obtained in terms of CYT accumulation into the buccal tissue. Indeed, the administration of 8, 10 and 15 mg/mL solutions led to a similar recovered amounts of CYT (≈500 µg/cm^2^), which is likely the maximum amount of drug that can remain entrapped, due to tissue saturation.

The obtained results highlighted the stability of CYT in buccal environmental conditions, as well as the capability of CYT to penetrate and permeate buccal tissue. These issues underline the real possibility of benefiting from the advantages of buccal administration, as a useful alternative to the currently per os smoking cessation therapy, by designing effective BDS.

### 4.2. Design, Preparation, and Characterization of CYT-Loaded Buccal Tablets

#### 4.2.1. Excipients Selection

Buccal drug delivery offers several advantages when compared to conventional oral administration, including rapid absorption, fast onset of action, avoidance of first pass effect metabolism and increased patient compliance and adherence to therapy [28]. As already mentioned, CYT is able to effectively cross the buccal mucosa and its disadvantages after oral administration, such as short half-life, significant first pass effect metabolism, tight therapeutic regimen requiring frequent and multiple administration make this molecule particularly suitable for buccal drug delivery. Among the many formulations used for buccal administration, solid dosage forms are generally preferable to liquid or semi-solid ones, due to their ability to allow longer in situ residence time, controlled and sustained drug release behavior, standardization of the administered dose and possibility of easy self-administration [29,30]. In recent years, matrix buccal tablets have emerged as innovative solid BDSs. Buccal tablets are prepared by direct compressing a polymer-based powder, designed to be placed on the buccal mucosa and intended for loco-regional or transmucosal drug delivery [31]. They are generally matrix-like systems, composed of a polymer-based structure suitable for obtaining a controlled drug release profile. The choice of adequate excipients is of crucial importance to design an effective BDSs with all the desired characteristics, such as controlled and reproducible drug release behavior and a maximized contact time with the application site, which could both result in higher patients compliance and promote the success of the therapeutic strategy. To construct the matrix-like skeleton of the desired CYT-loaded BDSs, Eudragit^®^ RS100 was chosen as the main polymer. Eudragit^®^ is a brand name for different kinds of polymers based on polymethacrylate copolymers, and widely used due to their ability to form matrix structures. In this work Eudragit^®^ RS100 was selected, since its cationic nature makes it mucoadhesive due to promoted interactions with negatively charged mucins [32]. Therefore, its ammino quaternary groups confer low water permeability and pH-independent swelling, resulting in a suitable to control drug release profile as well as to causing minimum patient discomfort [33]. It is well known that, by mixing two or more polymers it is possible to obtain a new material that has different and better mechanical, morphological and physico-chemical properties [34]. For this reason, hydrophilic PEG 1000 was chosen as an additional polymer, firstly to improve water entry into the matrix, allowing the CYT to discharge and, secondly, to enhance the mucoadhesiveness of the final dosage form [35]. Moreover, in order to further increase the mucoadhesive properties of the formulations, in some cases PVP-K90 was also added. This is a synthetic hydrophilic polymer widely used as the main compound for the preparation of pharmaceutical dosage forms, due to its excellent wetting and mucoadhesive properties [36]. Moreover, since the final dosage forms must be placed inside the oral cavity, their taste is an important parameter to consider. As saccharose is not suitable for people who are on a restricted diet or for diabetic patients, xylitol was chosen as an alternative sweetener. Xylitol is a sugar polyol that could also improve drug permeability, acting as permeation enhancer molecule. Finally, propylene glycol was also chosen as a hydrophilic excipient as it is able to promote buccal permeation and tissue accumulation of active compounds in association with polyols, thus making it indispensable for the preparation of solid buccal formulations [20,37].

#### 4.2.2. Optimization of the Spray-Drying Parameters

In this work, four powders (S-CYT-A, S-CYT-B, S-CYT-C and S-CYT-D) characterized by different excipient ratios were obtained by spray-drying. The spray-drying method was chosen since it is easily scalable, cost-effective, and valuable to obtain dry pharmaceutical powders that are homogeneous in terms of both particle size (narrow size distribution) and drug loading [20]. This process is characterized by some relevant parameters (e.g., type of solvent, flow rate, inlet temperature, starting solution concentration, spray-dried volume), which directly affect the characteristics of the final spray-dried product. While some of these parameters were fixed according to our previous work (starting solution concentration: 5% *w*/*v*; flow rate: 100 mL/h) [20], the type of solvent was the first investigated variable. The literature reports the use of methanol, ethanol and acetonitrile to obtain Eudragit^®^ RS100 spray-dried powder, and thus these three organic solvents were chosen, as well as because of their favorable boiling points and ability to dissolve hydrophilic excipients. Acetonitrile was immediately discharged as it was not able to completely dissolve xylitol, which is present in the composition of S-CYT-B, S-CYT-C and S-CYT-D. In contrast, ethanol and methanol allow suitable solutions to be quickly obtained, and they were consequently used for the preparation of the spray-dried powders. The second parameter investigated was the selected inlet temperature, which depends on the chosen solvent, as it generally must be almost 10 degrees above the boiling point in order to ensure the complete evaporation of the solvent. As a consequence, when spray-drying methanol solution, 75 °C was set, while for ethanol solution, the inlet temperature was 110 °C in order to also evaporate water content. As a result, the powders obtained by the ethanol solutions were in large grains, often compacted to form sheets, which were hard to manage since they were strongly adhesive and difficult to recover. This could be attributed to a small amount of unremoved water, making the final product extremely cohesive. On the other hand, the powders obtained by methanol solutions were easily recoverable, fine, and dry. Consequently, methanol was finally chosen to prepare the subsequently reported formulations. Once the process parameters were set, the four different powders were prepared and characterized. The four compositions differ in terms of excipient ratio and presence/absence of some selected components, in order to evaluate their contribution to some relevant characteristics (e.g., permeation enhancer effect, stability). All the powders are composed of Eudragit^®^ RS100, PEG1000 and CYT. S-CYT-A, S-CYT-B and S-CYT-C also contain propylene glycol; xylitol was added in S-CYT-B, S-CYT-C and S-CYT-D, while only S-CYT-C contains PVP K90.

#### 4.2.3. Spray-Dried Matrices Characterization and Tablets Preparation and Evaluation

As reported, the results highlighted optimal yield % values (above 50% *w*/*w*) for all the samples. According to the literature, a relevant disadvantage of the spray-drying process is product loss on the walls of the drying chamber that negatively affects the final yield, which generally falls into the 20–70% (*w*/*w*) range [38]. The stability of CYT in the experimental conditions, as well as the homogeneity of the obtained samples, were proved by evaluating the actual amount of CYT in the recovered powders both by UV-Vis and HPLC analyses. In particular, neither degradation peaks nor modification in terms of shape, retention time or new peaks were detected by the HPLC-DAD (200–700 nm range) studies, confirming that the preparation method does not affect the chemical structure of the drug. Furthermore, as HPLC and UV-Vis data were comparable in terms of calculated DL% values, for repeated batches of the same composition, the UV-Vis method was chosen to quickly analyze samples while also avoiding any waste of organic solvents. Moreover, these quantitative analyses were repeated on randomly selected aliquots of powders so that the DL% and LE% standard error values could be used as a proof of both sample homogeneity and reproducibility of the preparation method. To better characterize the spray-dried powders, FTIR and XRD analyses were performed. The FTIR analyses highlighted that interaction between CYT and Eudragit^®^ RS100 occurred. The FTIR spectra recorded for all the powders are characterized by the disappearance of CYT bands at 3278 and 3319 cm^−1^. As the HPLC-based DL% studies had already demonstrated that no CYT degradation phenomena occurred, FTIR analyses were conducted on both a physical CYT–Eudragit mixture and a spray-dried powder composed of only CYT and Eudragit. As a result, when subjected to spray-drying, CYT and Eudragit^®^ RS100 established an interaction, leading to the discussed modifications in terms of their FTIR spectra. In contrast, when they were physically mixed, no interactions were detectable. The drug’s physical state (crystalline or amorphous) could be extremely relevant in favoring several key processes e.g., solubilization, drug release and, consequently, permeation. At this point, the performed XRD studies highlighted the complete amorphization of all of the crystalline molecule inserted in the formulations (CYT and also xylitol when present). Furthermore, the ability of the whole excipients to prevent the re-crystallization of CYT during storage could be a key factor in the pharmaceutical field, since the amorphous form makes the drug more bioavailable and resistant to degradation phenomena. As the prepared powders seems to possess all the required characteristics, they were all used to prepare four different types of buccal tablets by direct compression. As reported, the reproducibility of the preparation method was proved by evaluating the following relevant parameters: drug content, thickness and weight, together with their standard errors. Results showed high morphological uniformity and homogeneous drug content for all the samples, making them suitable for further studies.

#### 4.2.4. CYT Release Studies and Evaluation of the Drug Discharge Mechanism

The first technological characterization we performed was about CYT release behavior, since the key factor to ensure drug transport though the target tissue is the capability of releasing compounds from the drug delivery system to the biological fluid. Usually, drug release studies are performed according to the pharmacopoeias that describe experiments performed in a high dissolution media volume under sink or pseudo-sink conditions to allow a complete release of the drug. In this work since the formulations would be administered on buccal tissue, which is characterized by a small amount of liquid and where sink conditions are often not reached, drug release studies were performed using a peristaltic pump and stimulated salivary fluid as a medium, in order to better mimic in vivo conditions [18]. The resulting data highlight that excipient composition, even when slight modifications occurred, directly affects the behavior of CYT discharge. All of the four formulations are able to release the total amount of loaded drug after 3 h, despite showing different starting rates of release for the different compositions. Xylitol and propylene glycol act in synergy as hydrophilic and fast dissolving excipients, thus speeding up the release rate (see S-CYT-B). When only one of them is present (propylene glycol in S-CYT-A and xylitol in S-CYT-D) a slowing down of the discharge pattern is observable, as well as the finding that the xylitol effect on the drug release rate is greater than that of propylene glycol. Finally, the insertion of PVP K90 (S-CYT-C) probably causes a general variation of the polymeric matrix arrangement, thus reducing the drug discharge, despite the combined effect of xylitol and propylene glycol. To better understand the drug release experiments, data were curve fitted by the most common mathematical models used in this field. The best fitting results were obtained with the Korsmeyer–Peppas (power law) and Peppas–Sahlin equations. However, it is noticeable that the application of the Korsmeyer–Peppas model gave anomalous n values (n < 0.5), which are probably related to a high prevalence of Fickian diffusion [39,40]. This is also supported by the curve fitting results obtained when applying the Peppas–Sahlin model. This equation starts from the power law model but considers the contribution of two different mechanisms (diffusion and relaxation/erosion) in an anomalous drug release process. The Peppas–Sahlin equation can be divided as follow: the first term represents the Fickian diffusional contribution (F), while the second term represents the Case II relaxational/erosional contribution (R). The proposed tablets are mainly composed of Eudragit^®^ RS100 which is a non-erodible polymer; however, it is likely to experience a sort of relaxation due to hydration as well as dissolution of the water-soluble components (xylitol, propylene glycol, PEG1000 and CYT). To better understand the driving force governing the discharge phenomenon, the F and R parameters were calculated. As reported, the R/F ratio decreases over time because the F contribution increases while the R decreases. This further confirms that the Fickian diffusion is the predominant mechanism of drug discharge. Furthermore, the evidence that the drug is released within 3 h when subjected to a continuous flow of saliva (a condition probably forced in the release test) suggests that the tablets are capable to produce a concentrated solution of CYT in close contact with the buccal mucosa for an extended time and are able to prolong drug absorption and consequently constant plasma levels, thus reducing the frequency of daily administration.

#### 4.2.5. Ex Vivo Tests and Pharmacokinetic Considerations

To demonstrate the ability of the buccal tablets to promote CYT permeation through the mucosa, ex vivo experiments were performed analogously to what has been done for the CYT solutions. It was found that, for improving CYT accumulation into the target tissue, the presence of propylene glycol is fundamental, due to its ability to interact with the hydrophilic domains of the mucosa [41]. Indeed, the sample prepared without propylene glycol (S-CYT-D) shows the lowest De and Ac values. The drug amount trapped in the mucosa after tablet administration also shows that the membrane is still far from saturation, despite the drug flux values being markedly high indicating an increased CYT permeability.

However, with regard to buccal administration to achieve systemic effects the main parameters to be considered are those related to CYT permeability through the mucosa. In particular, considering a technological point of view, the encapsulation of CYT into a matrix system positively affects its permeability, as the calculated Kp values increased by an order of magnitude referred to as the intrinsic Kp value of CYT (see above). On the other hand, considering a pharmacological point of view, the actual Js values obtained by administering the matrix tablets should be considered and might be found to be correlated with the existing pharmacokinetic knowledge on CYT. These pharmacokinetic considerations could prove the great potential and usefulness of the proposed tablets as an alternative therapeutic strategy, instead of the currently available oral treatment.

The following parameters, related to a single oral administration of 1.5 mg of CYT (conventional treatment) might be considered:

- C_max_: 12.1 ± 2.2 ng/mL;
- t_1/2_: 4.4 ± 0.5 h (as a consequence Ke = 0.693/t_1/2_ = 0.1575 h^−1^);
- V_D_: 110.1 ± 19.0 L [9].

Due to the high elimination rate of CYT, the steady state conditions are not available and repeated administrations aimed at maintaining the C_max_ are necessary. As a consequence, the C_max_ value will be used here as a target concentration to be constantly reached, instead of the generally employed steady state concentration value. To achieve these ideal steady state conditions, the drug absorption rate (DR_A_) must be equal to the drug elimination rate (DR_E_) and thus, considering the V_D_, it is possible to calculate the amount of CYT per hour that has to be absorbed to maintain a constant and effective plasma concentration, as follows:

(10) $${\mathrm{DR}}_{E}=\frac{C_{max}\;}{K_{e}}\left(\mathrm{ng}/\mathrm{mL}\;\cdot \;h^{-1}\right)$$

(11) $${\mathrm{DR}}_{A}=\frac{{\mathrm{DR}}_{E}}{V_{D\;}}\left(µg/h\right)($$

In particular, DR_E_ results in 1.91 ng/(mL ∙ h^−1^), and considering the V_D_ for an average 70 kg adult it is possible to calculate the required DR_A_ which is equal to 210 µg/h.

The application of the BDS in the buccal mucosa (e.g., middle third in the medio-distal direction and caudal third in the cranio-caudal direction, in an area less involved in mandibular movements) could be strategic, since drug release and permeation phenomena will occur in two directions, all along the tablet surface. Moreover, this anatomical region is characterized by the presence of a low volume of saliva, preventing the loss of a large amount of drug due to swallowing. Moreover, since CYT flux through the mucosa directly depends on the contact surface, it should be possible to adjust and modulate the diameter of the administered tablet, in order to reach the inlet drug rate needed to establish effective steady state conditions. In addition, these steady state conditions are maintained for a prolonged time, determined by the control of drug release operated by the matrix tablets.

For pharmacokinetic consideration of the ex vivo data, some issues should be firstly pointed out. The reported Js values are normalized to the surface and consequently referred to 1 cm^2^. They were calculated by considering the whole surface of the Franz cell as area of permeation, as this phenomenon could occur both at the interface between the tablet and the tissue and at the fluid–mucosa interface (as CYT is a water-soluble drug, thus creating a CYT-loaded solution in the donor chamber). Since it is not possible to calculate the in vivo actual volume of saliva, or of the whole surface of application, it could be better to base the flux considerations only on the area occupied by the buccal tablet. Assuming negligible tablet thickness, the actual contact area for the prepared BDS is 0.66 cm^2^, which is the sum of the two surfaces of the tablet. Knowing the actual exchange area for each tablet, it is possible to calculate the drug absorption rate and consequently, by fixing the desired DR_A_ value, the theoretical dimension of the tablet to be administered is obtainable (Table 6). It is worth underlining that these approximations are underestimated, as some relevant parameters cannot not be taken into account. In particular, the volume of saliva is not considered and, consequently, the real exchange area is not known (tablet–mucosa and CYT-enriched saliva surfaces). Furthermore, by increasing the diameter of the administered tablet, the starting CYT dose can also be enhanced.

Finally, it is interesting to observe the ex vivo data obtained in terms of drug accumulation into the tissue. Indeed, it is noticeable that the S-CYT-A and S-CYT-B formulations result in De values comparable to those obtained after the application of 2 and 5 mg/mL CYT solutions, respectively, although the real drug concentration in the donor chamber is lower when administering the BDSs. Additionally, the obtained De value for all the designed formulations is still very far from the values obtainable when membrane saturation occurs. Combining the ex vivo results (a significant increase in terms of Js and Kp, while De is lower than that related to tissue saturation), demonstrates the great permeation enhancer effect of all the proposed BDSs. Last but not least, the t_lag_ required to achieve the steady state conditions should also be considered. The currently available CYT-based smoking cessation therapy leads to the C_max_ after 2 h of administration. In contrast, the BDSs proposed in this study are characterized by lag times ranging from 23 to 70 min. Consequently, the effective plasma concentration might be achieved after a shorter time interval via buccal administration, and this could be particularly useful when craving symptoms appear.

## 5. Conclusions

The major aim of this work was the development of novel CYT-loaded BDSs to benefit from the buccal route of administration in smoking cessation therapy, thus avoiding the disadvantages related to the conventional oral route while also improving patient compliance and adherence to therapy. CYT emerged as a perfect candidate to be administered in the oral cavity and to obtain transmucosal absorption, due to its water-solubility, stability in the environmental conditions of the mouth and high permeability of the buccal tissue. Considering the advantages of a solid dosage form to be applied on the buccal mucosa, this work proposes the preparation of buccal sustained-release matrix tablets. To efficiently prepare the powders to be compressed, a spray-drying technique was chosen as it is a rapid, effective, scalable and reproducible process. Several process parameters were investigated to identify the best conditions and achieve good powders in terms of reproducibility, recovery and workability. Four different powder compositions were prepared by using Eudragit^®^ RS100 as the main polymer, PEG1000 as a copolymer and varying the other additives, to confer some relevant ad hoc characteristics. The four powders were directly compressed, thus obtaining reproducible buccal tablets that were evaluated in terms of drug discharge behavior and permeation performance. The in vitro drug release profiles highlighted the ability to completely discharge the loaded CYT by a diffusive Fickian mechanism, due to the inert Eudragit-based matrices. The presence of crucial hydrophilic excipients (e.g., xylitol, propylene glycol) or the insertion of secondary polymers (e.g., PVP K90) resulted in the ability to modulate the rate of CYT release, while maintaining the fitting to the Korsmeyer–Peppas and Peppas–Sahlin kinetic models. Finally, the ex vivo studies highlighted a significant permeation enhancer effect (the CYT Kp value is increased by an order of magnitude for all the formulations), resulting in drug fluxes that are potentially useful to achieve the same plasmatic concentration as that reported after a single oral dose of 1.5 mg (conventional treatment) and to maintain it over time. These results are very promising and make these buccal delivery systems very valuable candidates for further in vivo studies, suggesting their potential effectiveness as an efficient alternative to the current oral therapy for smoking cessation, both in terms of efficacy and patient compliance and adherence to treatment during symptoms of craving.

## Figures and Tables

**Table 1:** Percentage composition (*w*/*w*) of CYT-loaded matrices prepared by a spray-drying technique.

	S-CYT-A	S-CYT-B	S-CYT-C	S-CYT-D
**Eudragit^®^ RS100**	75	70	65	80
**PVP K90**	-	-	5	-
**PEG 1000**	10	10	10	10
**Propylene Glycol**	10	10	10	-
**Xylitol**	-	5	5	5
**Cytisine**	5	5	5	5

**Table 2:** CYT biopharmaceutical parameters calculated by the ex vivo evaluations: Js (µg/cm^2^ ∙ h^−1^), Kp (cm/h), De (µg/cm^2^), Ac (cm) and t_lag_ (min) ± SE (n = 6).

CYT (mg/mL)	Js (µg/cm^2^ ∙ h^−1^)	Kp (cm/h)	De (µg/cm^2^)	Ac (cm)	t_lag_ (min)
**2**	4.94 ± 0.16	0.00247 ± 0.00001	164.72 ± 13.12	0.08236 ± 0.00656	-
**5**	21.02 ± 4.69	0.00420 ± 0.00094	171.86 ± 17.52	0.02186 ± 0.00223	60
**8**	36.17 ± 5.40	0.00452 ± 0.00068	511.30 ± 95.02	0.04065 ± 0.00755	90
**10**	46.77 ± 6.18	0.00468 ± 0.00062	559.30 ± 93.66	0.03557 ± 0.00596	100
**15**	45.62 ± 3.66	0.00304 ± 0.00024	524.64 ± 33.45	0.02225 ± 0.00142	110

**Table 3:** Characterization of the CYT-loaded spray-dried powders (yield %, DL% and LE% ± SE; n = 3) and CYT-loaded buccal tablets (thickness, weight, and drug content ± SE; n = 9).

	Powders			Tablets		
Composition	Yield %	DL %	LE %	Thickness (mm)	Weight (mg)	CYT Per Tablet (mg)
**S-CYT-A**	63.0 ± 1.0	5.12 ± 0.02	102.4 ± 0.4	1.01 ± 0.03	18.00 ± 0.94	0.92 ± 0.05
**S-CYT-B**	63.0 ± 3.5	5.84 ± 0.48	116.8 ± 9.6	1.05 ± 0.01	17.24 ± 1.25	1.07 ± 0.08
**S-CYT-C**	53.7 ± 5.0	5.41 ± 0.12	108.2 ± 2.4	0.91 ± 0.04	17.29 ± 0.76	0.99 ± 0.04
**S-CYT-D**	76.0 ± 0.1	4.72 ± 0.01	94.4 ± 0.2	0.88 ± 0.02	20.51 ± 1.26	0.97 ± 0.06

**Table 4:** Mathematical models fitted to the experimental release curves: calculated fitting parameters and square of coefficient of determination (R2). Data were fitted until the 90% of CYT was released (75, 40, 50 and 55 min for S-CYT-A, S-CYT-B, S-CYT-C and S-CYT-D respectively).

Mathematical Model	S-CYT-A	S-CYT-B	S-CYT-C	S-CYT-D
**Zero order** ***D = k·t***	k = 0.01485 ± 0.00103 R = 0.472	*Does not converge*	*Does not converge*	*Does not converge*
**First order** ***D = 1·(1 − e^k·t^)***	k = 0.03675 ± 0.00116 R = 0.982	k = 0.07436 ± 0.00653 R = 0.889	k = 0.07824 ± 0.00262 R = 0.896	k = 0.05376 ± 0.00222 R = 0.953
**Higuchi** ***D = k·t*** **^0.5^**	k = 0.09050 ± 0.00176 R = 0.743	k = 0.15658 ± 0.00445 R = 0.863	k = 0.16270 ± 0.00527 R = 0.705	k = 0.13514 ± 0.00285 R = 0.909
**Korsmeyer–Peppas (Power Law)** ***D = k·t^n^***	k = 0.23273 ± 0.02238 n = 0.2996 ± 0.02048 R = 0.931	k = 0.25748 ± 0.02174 n = 0.34963 ± 0.02554 R = 0.974	k = 0.24749 ± 0.01109 n = 0.34487 ± 0.01665 R = 0.974	k = 0.20754 ± 0.01829 n = 0.38014 ± 0.02458 R = 0.971
**Hixson–Crowell** ***D = 1·[1 − (1 − k·t)*** **^3^** ***]***	k = 0.00991 ± 0.00065 R = 0.905	k = 0.01865 ± 0.00186 R = 0.703	k = 0.02257 ± 0.00104 R = 0.746	k = 0.01389 ± 0.00090 R = 0.834
**Peppas–Sahlin** ***D = k*** **_1_** ***·t^m^ + k_2_·t*** **^2*m*^** (m = 0.43) *	k_1_ = 0.014941 ± 0.01126 k_2_ = −0.00028 ± 0.00206 R = 0.974	k_1_ = 0.25062 ± 0.01200 k_2_ = −0.01263 ± 0.00283 R = 0.985	k_1_ = 0.23389 ± 0.00530 k_2_ = −0.01117 ± 0.00155 R = 0.985	k_1_ = 0.20417 ± 0.01072 k_2_ = −0.00645 ± 0.00224 R = 0.978

* the *m* value was determined as reported in the literature [24] by calculating the aspect ratio of each formulation (diameter/thickness).

**Table 5:** CYT biopharmaceutical parameters calculated by the ex vivo evaluations: Js (µg/cm^2^ ∙ h^−1^), Kp (cm/h), De (µg/cm^2^), Ac (cm) and t_lag_ (min) together with CYT experimental concentration into the donor chamber ± SE (n = 6).

Formulation	Js (µg/cm^2^ ∙ h^−1^)	Kp (cm/h)	De (µg/cm^2^)	Ac (cm)	[CYT]_DONOR_ (mg/mL)	t_lag_ (min)
**S-CYT-A**	21.11 ± 2.86	0.01939 ± 0.00319	164.44 ± 28.73	0.15107 ± 0.02437	1.09 ± 0.09	69
**S-CYT-B**	32.40 ± 0.63	0.03417 ± 0.00712	181.96 ± 12.22	0.19192 ± 0.04084	0.95 ± 0.10	23
**S-CYT-C**	18.05 ± 2.80	0.01608 ± 0.00279	98.23 ± 13.87	0.08753 ± 0.01374	1.12 ± 0.03	70
**S-CYT-D**	18.77 ± 3.71	0.01240 ± 0.00290	94.45 ± 11.46	0.06321 ± 0.00480	1.40 ± 0.27	50

**Table 6:** Speculative pharmacokinetic considerations: effective surface of tested tablets and calculated surface of tablets to obtain a DR_A_ of 210 µg/h.

Formulation	CYT Flux	Tested Tablet Parameters	Theoretical Tablet Parameters
**S-CYT-A**	21.11 µg/cm^2^ ∙ h^−1^	DR_A_: 13.93 µg/h Total exchange area: 0.66 cm^2^ Diameter: 0.65 cm	Total exchange area: 9.95 cm^2^ Diameter: 2.52 cm
**S-CYT-B**	32.40 µg/cm^2^ ∙ h^−1^	DR_A_: 21.38 µg/h Total exchange area: 0.66 cm^2^ Diameter: 0.65 cm	Total exchange area: 6.48 cm^2^ Diameter: 2.03 cm
**S-CYT-C**	18.05 µg/cm^2^ ∙ h^−1^	DR_A_: 11.91 µg/h Total exchange area: 0.66 cm^2^ Diameter: 0.65 cm	Total exchange area: 11.63 cm^2^ Diameter: 2.72 cm
**S-CYT-D**	18.77 µg/cm^2^ ∙ h^−1^	DR_A_: 12.39 µg/h Total exchange area: 0.66 cm^2^ Diameter: 0.65 cm	Total exchange area: 11.19 cm^2^ Diameter: 2.67 cm
